# The Combined Model of CX3CR1-Related Immune Infiltration Genes to Evaluate the Prognosis of Idiopathic Pulmonary Fibrosis

**DOI:** 10.3389/fimmu.2022.837188

**Published:** 2022-02-10

**Authors:** Haozheng Cai, Shijie Chen, Xinyu Li, Hanying Liu, Ying Zhang, Quan Zhuang

**Affiliations:** ^1^ Transplantation Center, The 3rd Xiangya Hospital, Central South University, Changsha, China; ^2^ Department of Spine Surgery, The 3rd Xiangya Hospital, Central South University, Changsha, China; ^3^ Xiangya School of Medicine, Central South University, Changsha, China; ^4^ Department of Respiratory Diseases, The 3rd Xiangya Hospital, Central South University, Changsha, China; ^5^ Research Center of National Health Ministry on Transplantation Medicine, Changsha, China

**Keywords:** CX3CR1, idiopathic pulmonary fibrosis, immune infiltration, prognosis, activated NK cell

## Abstract

**Background:**

High expression of chemokine (C-X3-C motif) receptor 1 (CX3CR1) was shown to contribute to the progression of many fibrotic diseases. However, there is still no study for the role of CX3CR1 in idiopathic pulmonary fibrosis (IPF). Therefore, we aimed to identify CX3CR1-related immune infiltration genes (IIGs) in IPF and establish a combined risk model to evaluate the prognosis of IPF.

**Methods:**

A discovery cohort of IPF patients (GSE70867) was downloaded from the Gene Expression Omnibus dataset. We identified the composition of 22 kinds of immune cells infiltration by CIBERSORT. The Cox regression model with the LASSO method was used for identifying prognostic genes and developing CX3CR1-related IIGs. Kaplan–Meier was applied to plot the survival curve of prognosis model. Peripheral blood mononuclear cell (PBMC) and bronchoalveolar lavage fluid (BALF) were collected to be tested by quantitative reverse transcriptase-PCR (qRT-PCR) from 15 clinical samples, including 8 healthy controls (HC), 4 patients with usual interstitial pneumonia (UIP) and 3 patients with pulmonary fibrosis (FIB).

**Results:**

We found that high expression of CX3CR1 in BALF contributed to the poor prognosis in IPF patients. ALR4C, RAB37, GPR56, MARCKS, PXN and RASSF2 were identified as CX3CR1-related IIGs, which were highly expressed in PBMC of UIP/FIB patients than that of HC. Moreover, the expression of PXN was higher in FIB patients’ PBMC than that of UIP ones. In the cohort of IPF patients, high infiltration of activated NK cells in BALF caused poor survival compared to low infiltration group. The infiltration of activated NK was regulated by CX3CR1-related IIGs. The combined risk model predicted that high expression of CX3CR1-related IIGs and high infiltrated activated NK cells caused poor prognosis in IPF patients.

**Conclusion:**

We identified a group of CX3CR1-related IIGs in IPF patients. This combined risk model provided new insights in the prognosis and therapy of IPF.

## Introduction

Idiopathic pulmonary fibrosis (IPF) is a consequence of recurrent microinjuries to alveolar epithelium cells, triggering an abnormal fibrotic response in the lung parenchyma of a genetically susceptible individual (1). Healthy tissue is displaced by altered extracellular matrix and alveolar architecture is destroyed, which contributes to decreased lung compliance, influenced gas exchange, and ultimately respiratory failure and death. The morbidity of IPF in patients (>) has reached 400 cases per 10,000 people, and the incidence of IPF is increasing as age goes by (2). Except lung transplantation, the current therapeutic methods for IPF are still lacking and insufficient. Therefore, novel drug targets for the prevention and treatment of IPF progression are urgently needed.

Chemokine (C-X3-C motif) Receptor 1 (CX3CR1) is mainly expressed on most of myeloid cells (3) and combined with its only ligand CX3CL1 to mediate chemotaxis and adhesion of immune cells (4). The role of CX3CR1 in the progression of organ fibrosis is still controversial. Several studies showed that high expression of CX3CR1 resulted in the exacerbation of obstruction-induced renal fibrosis and bleomycin-induced pulmonary fibrosis (3, 5). However, there were also some studies which demonstrated that high expression of CX3CR1 might play a protective role in kidney and liver fibrosis (6, 7). At present, few studies are acquainted for the role of CX3CR1 in the predictive outcome of IPF.

The degree of immune cell infiltration in peripheral blood mononuclear cell (PBMC) and bronchoalveolar lavage fluid (BALF) also has an important impact on IPF progression. In our previous studies, we found that patients with high immune cell infiltration in BALF led to poor survival compared to low infiltration ones. High levels of activated NK cells, M1 macrophages, activated mast cells, activated CD4**^+^** memory T cells and CD8**^+^** T cells are risk factors for the development of IPF. However, high infiltration of M0 macrophages plays a protective role in IPF patients (8, 9). As we described before, CX3CR1 mainly mediated chemotaxis, migration, and adhesion of immune cells; thus, we speculated that the expression of CX3CR1 may be associated with immune cell infiltration. In a recent study, Satoshi et al. demonstrated that CX3CR1 level was significantly higher in M1 macrophages of BALF in interstitial lung disease of mice. When the expression of CX3CR1 was inhibited in BALF, the infiltration of M1 macrophages decreased (10). Therefore, to study the changes and invasion of immune cells mediated by CX3CR1 in the process of pulmonary fibrosis is very helpful for the treatment of IPF.

In this study, we innovatively explored the network of CX3CR1-related immune infiltration genes (IIGs) of bronchoalveolar lavage fluid (BALF) cells in IPF patients. We also collected clinical samples from usual interstitial pneumonia (UIP) and patients with pulmonary fibrosis (FIB) patients to verify our bioinformatic findings. Our study demonstrates that CX3CR1 may become the new target for the prevention and treatment of IPF progression in the future.

## Materials and Methods

**Figure 1:**
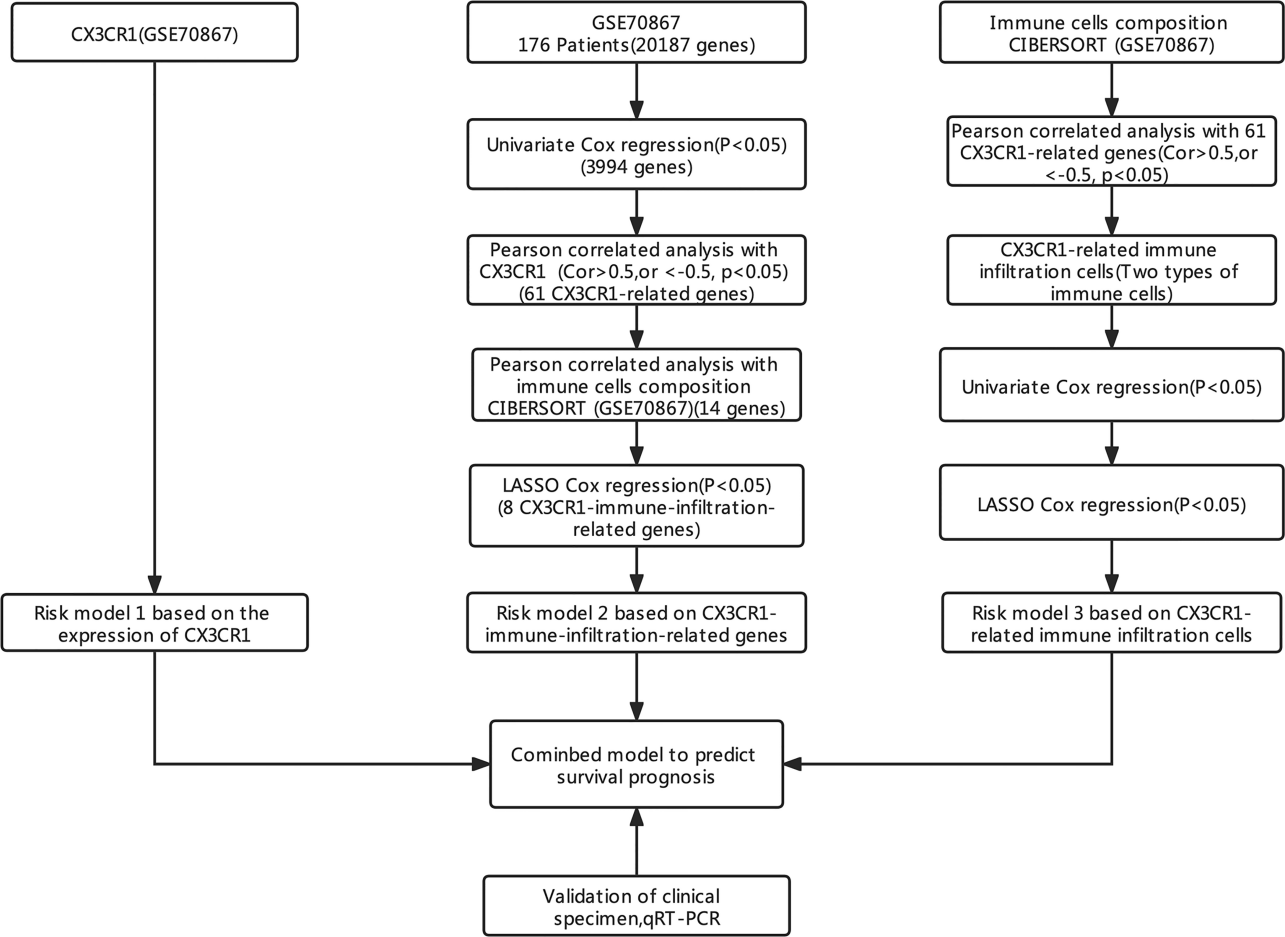
The research ideas and analysis process.

The process and methodologies of bioinformatic analysis used in this study are described in **Figure 1**.

### Preparation of Discovery Set

The discovery set was downloaded from https://www.ncbi.nlm.nih.gov/geo/(GSE70867), and this data set consisted of 176 IPF patients. The results of whole-genome sequencing came from the BALF cells of these patients. The information of these patients are presented in **Table 1**.

**Table 1:** The clinical information of patients (GSE70867).

Characteristics (GSE70867) ALL (n = 176 patients)	
**Age n (%)**	
**＞65 Years**	86 (48.9)
**＜65 Years**	90 (51.1)
**Gender n (%)**	
**Male**	117 (66.5)
**Female**	59 (33.5)
**Survival n (%)**	
**Live**	76 (43.2)
**Dead**	100 (56.8)
**Survival time (days)**	699.3 ± 518.6

### Identify Whether CX3CR1 is a Risk Factor in IPF

**Figure 2:**
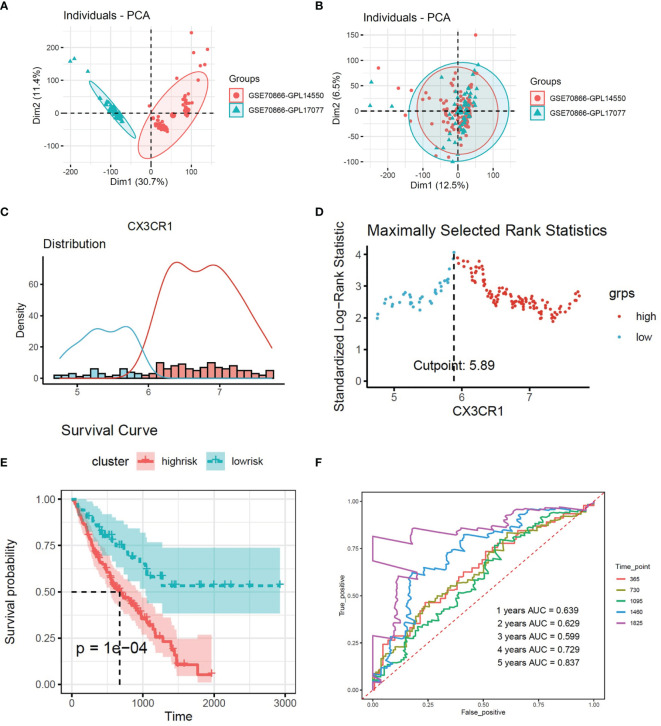
**(A, B)** Removing the batch effect between different sequencing platforms. **(C)** Histogram based on maximally selected rank grouping. **(D)** The cut-off point with the maximum standard log-rank statistic was marked with a vertical dashed line. **(E)** Kaplan–Meier plot of overall survival in two clusters based on the risk models of CX3CR1(Risk score1). **(F)** The ROC curve in the risk models of CX3CR1(Risk score1).

Firstly, sva package was used to avoid the batch impact (**Figures 2A, B**). We extracted the matrix of CX3CR1 expression from the discovery set, and the maximally selected rank statistics was used to divide patients into two groups: high and low risk. The high-risk group was characterized by high expression of CX3CR1. This algorithm was performed by R package “survival” and “survminer”. Combined with the clinical information, the Kaplan–Meier method was used to make survival plot create risk model 1, and the receiver operating characteristic (ROC) curve of this risk model was drawn to evaluate the sensitivity and specificity.

### Identification of Immune Infiltration Patterns in IPF Patients

In this study, the Estimation of Stromal and Immune cells in MAlignant Tumors using Expression data (ESTIMATE) algorithm was used to evaluate the immune infiltration of the BALF of the IPF patients (11). Maximally selected rank statistics was applied again to divide patients into two clusters (High- and low-immune groups). The corresponding survival curve was made to study the impact of different immune states to the survival of the patients. Meanwhile, CIBERSORT was used to obtain the composition of 22 types of immune cells in BALF of IPF patients. Only samples with CIBERSORT output P <0.05 were considered for further analysis.

### Identification of CX3CR1-Related Genes in IPF Patients

To further obtain genes related to the progression of the disease, univariate Cox analysis was used in the whole genome, and genes with p <0.05 were retained. Pearson correlation analysis was applied to identify genes that were correlated to CX3CR1 (with the | Pearson R | >0.5 and p <0.05). Then gene ontology functional annotation analysis (GO) and Kyoto Encyclopedia of Genes and Genomes analysis (KEGG) were used to identify the function and signal pathway of CX3CR-related genes. The analyses of GO and KEGG were performed in https://david.ncifcrf.gov/home.jsp

### Identification of the CX3CR1-Immune-Infiltration-Related Gene in IPF Patients

Pearson correlation analysis was applied to select genes which was related to immune cell infiltration from CX3CR1-related genes (with the | Pearson R | >0.5 and p <0.05). These genes were identified as CX3CR1-related immune infiltration genes (IIGs). Then the least absolute shrinkage and selection operator (LASSO) which is a type of linear regression using shrinkage was used to retain the optimal genes. Multivariate Cox regression was used to calculate the corresponding coefficients. The risk score of CX3CR1-related IIGs was calculated by Score = Σi = 1nCoefi ∗ the expression of relative gene. If the coefficient is greater than 0, it means that the gene is a risk factor. However, if the coefficient is less than 0, it means that the gene is a protective factor. Based on the risk score, Maximally selected rank statistics was used to split patients into two clusters. The survival curve and ROC curve were applied to predict the difference of survival condition between two clusters (Risk model 2).

### Identification of the CX3CR1-Related Immune Infiltration Cells in IPF Patients

We obtained the composition of 22 types of immune cells in BALF after CIBERSORT analysis. However, only immune cells which were correlated to CX3CR1-related genes were retained. These cells were identified as CX3CR1-related immune infiltration cells. According to the infiltration of retained immune cells, Maximally selected rank statistics was used to divide patients into high- and low-immune infiltration clusters, respectively. The survival curve and ROC curve were used to identify the impact of CX3CR1-related immune infiltration cells to survival time in IPF patients (Risk model 3).

### Prediction Model of IPF Prognosis Based on CX3CR1-Related IIGs and Immune Infiltration Cells

Based on the risk score of CX3CR1, CX3CR1-related IIGs and CX3CR1-related immune infiltration cells, the prediction model of IPF prognosis was established. The combined score was performed by Score = Σi = 1nCoefi ∗ corresponding risk score. The coefficients were calculated by Multivariate Cox regression. According to the combined risk score, Maximally selected rank statistics was applied to divide patients into high- and low-risk groups. Survival curve was made by the Kaplan–Meier method. ROC curve was drawn to evaluate the clinical influence of combined risk model.

### Verification of Clinical Samples

To verify the bioinformatic findings above, clinical samples were collected to test the expression of corresponding genes. Patients who were treated with corticosteroids and/or immunosuppressive agents were excluded from this study. The clinical samples were derived from 8 healthy controls and 7 patients. Seven patients consisted of 4 patients with UIP and 3 patients with FIB. The basic and clinical information of these patients are presented as **Table 2**. Our study was reviewed and approved by the institutional review board (Ethics Committee) of the 3rd Xiangya Hospital, Central South University (No. 21028). Lymphocytes Separation Medium was used to extract lymphocytes from peripheral blood. Quantitative reverse transcriptase-PCR (qRT-PCR) was applied to detect the expression of CX3CR1-related IIGs. TRIzol Reagent was used to extract the RNA from PBMC. Thermo Scientific PikoReal PCR cycler was applied to test RNA expression. Every gene was performed for three times and calculated for mean cycle threshold (CT) data. The equation 2^−△CT^ was calculated as relative gene expression.

**Table 2:** The clinical information of collect samples.

Characteristics	All (n = 7)	UIP (n = 4)	FIB (n = 3)
Age (>65), n (%)	2 (28.6%)	1 (25.0%)	1 (33.3%)
Male, n (%)	4 (57.1%)	2 (50%)	2 (66.7%)
A_aDO2 (mmHg)	140.9 ± 72.9	166.8 ± 75.5	115.0 ± 55.9
Hospital stay period (days)	9.1 ± 5.1	10.5 ± 6.2	7.3 ± 1.7

UIP, Usual interstitial pneumonia; FIB, Pulmonary fibrosis.

### Statistical Analysis

All analyses were performed with R version 4.0.2 and Graphpad Prism 9.0. Maximally selected rank statistics algorithm and survival plot were performed by R package “survival” and “survminer”. Immune score was performed by using R package “estimate”. CIBERSORT analysis was performed in https://cibersort.stanford.edu/. The LASSO and Cox regression model were performed by R package “glmnet”. ROC analysis was performed by R package “survivalROC” and “plotROC”. The results of PCR were presented as the mean ± standard deviation (SD). Continuous data were compared using the unpaired t-test or Mann–Whitney U test.

## Results

### High Expression of CX3CR1 in BALF Predicted Poor Prognosis in IPF Patients

According to the expression of CX3CR1, 176 patients were divided into two groups (**Figures 2C, D**). There were 125 patients in high-risk group and 51 patients in low-risk group. The survival curve showed that high expression of CX3CR1 in BALF could predict lower survival period in IPF patients compared to the low-risk group (**Figure 2E**). The area under the curve (AUC) values in the fourth and fifth years is greater than 0.7 (**Figure 2F**).

### CX3CR1-Related Gene in IPF Patients

**Figure 3:**
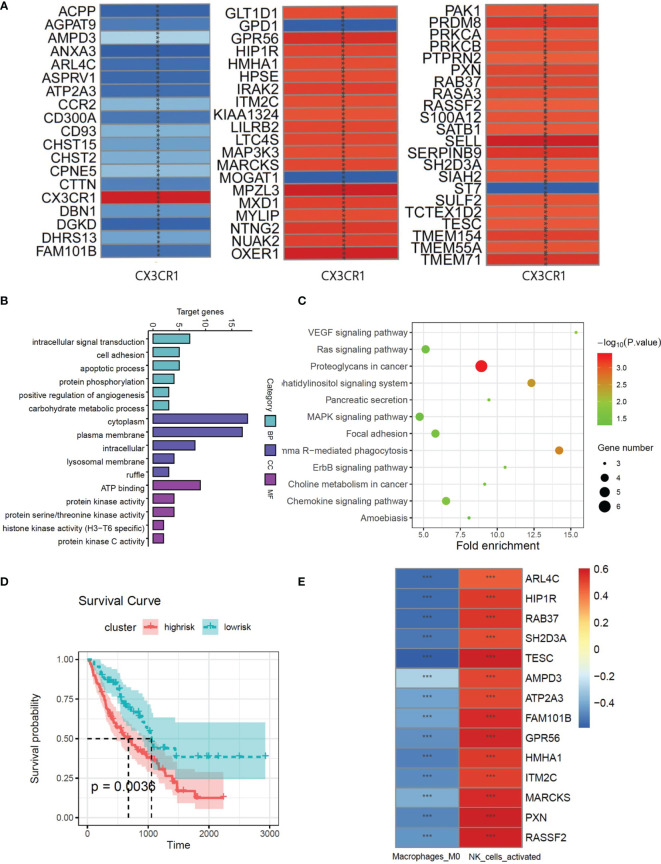
**(A)** The correlations between the CX3CR1 and genes which screened by univariate Cox analysis from whole genome. **(B)** GO function enrichment histogram of CX3CR1-related genes. **(C)** KEGG pathway bubble diagram of CX3CR1-related genes. **(D)** Kaplan–Meier plot of overall survival in two clusters based on the risk models of ESTIMATE immune score. **(E)** The correlations between the CX3CR1-related genes and 2 immune cell compositions. *** means P < 0.01.

A total of 3,994 genes were selected from 20,187 genes by univariate Cox regression. A total of 61 genes were screened from 3,994 genes by Pearson correlation analysis, which were identified as CX3CR1-related genes. The correlation heatmap is shown as **Figure 2A**. 61 CX3CR1-related genes were analyzed by gene ontology functional annotation analysis. CX3CR1-related genes were mainly concentrated on intracellular signal transduction, cell adhesion, and apoptotic process by biological process analysis. Cellular component analysis demonstrated that CX3CR1-related genes were enriched in cytoplasm, plasma membrane and intracellular. Molecular function analysis suggested that these genes had a great significance with ATP binging, protein kinase activity and protein serine/threonine kinase activity (**Figures 3A, B**). KEGG pathway analysis suggested that these genes were mainly enriched in proteoglycans in cancer, Fc gamma R-mediated phagocytosis, phosphatidylinositol signaling system and chemokine signaling pathway (**Figure 3C**).

### CX3CR1-Related Immune Infiltration Genes in IPF Patients

**Figure 4:**
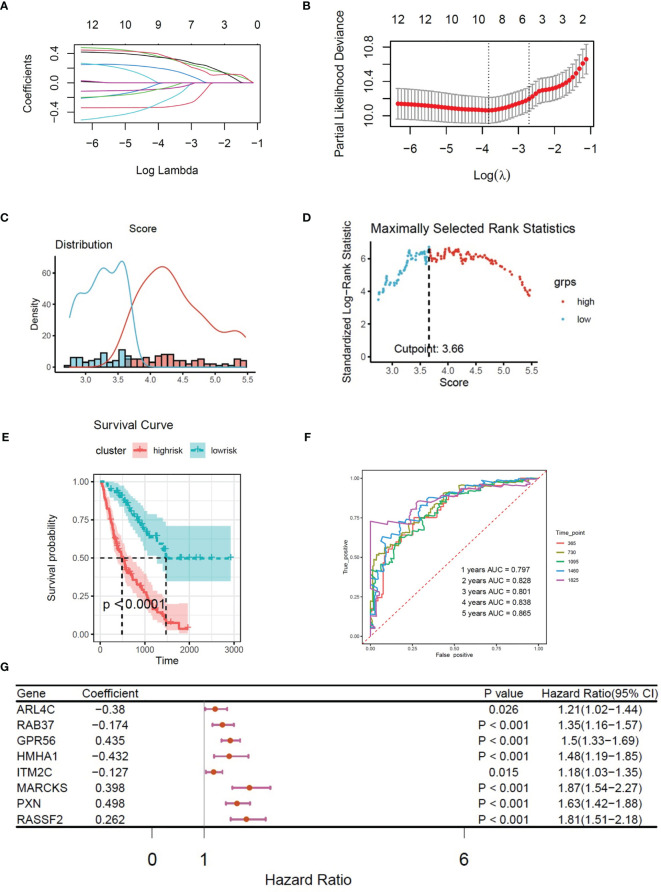
**(A)** LASSO coefficient profiles of 8 CX3CR1-related immune infiltration genes. **(B)** Three-fold cross-validation of lasso analysis. Error bars represented the SE. The dotted vertical lines showed the optimal values. **(C)** Histogram based on maximally selected rank grouping. **(D)** The cut-off point with the maximum standard log-rank statistic was marked with a vertical dashed line. **(E)** Kaplan–Meier plot of overall survival in two clusters based on the risk models of CX3CR1-related immune infiltration genes (Risk score2). **(F)** The ROC curve in the risk models of CX3CR1-related immune infiltration genes (Risk score2). **(G)** Forest plot of 8 CX3CR1-related immune infiltration genes with P < 0.05 by univariate Cox regression.

Multivariate Cox regression was used to evaluate the influence of the immune state of the patients to the disease severity. There were 66 patients in high-risk group and 110 patients in low-risk group. The survival plot shows that high immune state contributes to poor prognosis in IPF patients (**Figure 3D**). To study the immune infiltration in BALF, CIBERSORT was used to identify the composition of 22 types of immune cells. Fourteen genes were screened from 61 CX3CR1-related genes by Pearson correlation analysis. The correlation between CX3CR1-related genes and immune cells infiltration are presented in **Figure 2E**. Eight optimal prognostic genes were retained by LASSO regression (**Figures 4A, B**), which were identified as CX3CR1-related IIGs.

**Figure 5:**
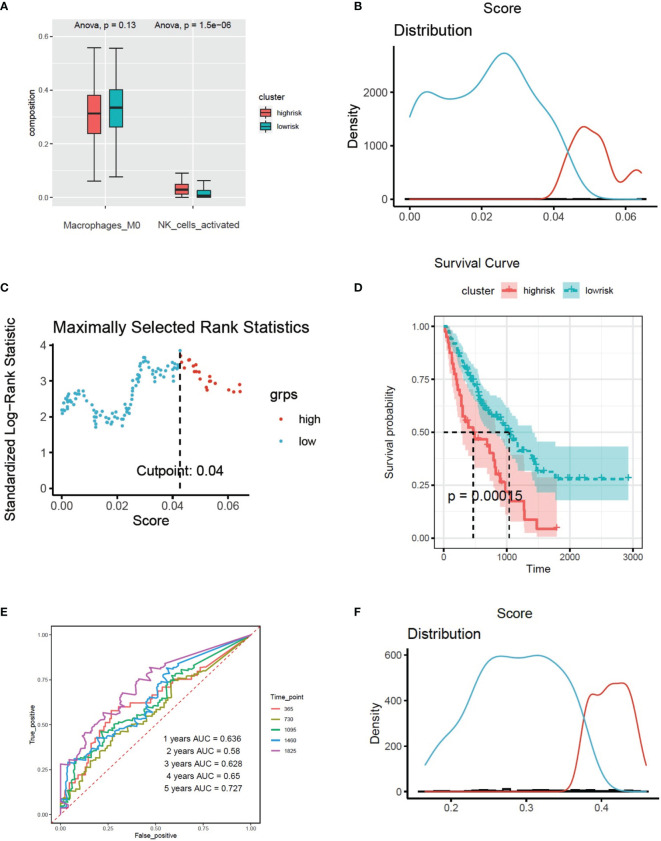
**(A)** The infiltration of activated NK cells and M0 cells in two clusters based on the risk models of CX3CR1-related immune infiltration genes (Risk score2). **(B)** Histogram based on maximally selected rank grouping. **(C)** The cut-off point with the maximum standard log-rank statistic was marked with a vertical dashed line. **(D)** Kaplan–Meier plot of overall survival in two clusters based on the infiltration of activated NK cells (Risk score3). **(E)** The ROC curve in the risk models of activated NK cells (Risk score3). **(F)** Histogram based on maximally selected rank grouping.

Based on the expression of 8 CX3CR1-immune-infiltration-related genes in the discovery set and their corresponding coefficients respectively, we calculated risk score 2 based on the formula: expression of ARL4C × (−0.3798) + expression of GPR56 × 0.4352 + expression of HMHA1 × (−0.4321) + expression of ITM2C × (−0.1269) + expression of MARCKS × 0.3978 + expression of PXN × 0.4978 + expression of RAB37 × (−0.1745) + expression of RASSF2 × 0.2619. Based on risk score 2, the maximally selected rank method was applied to divide patients into high-risk group (102 patients) and low-risk group (72 patients) (**Figures 4C, D**). Combined with survival information, Kaplan–Meier plot of overall survival in two groups indicated that the high-risk group had bad prognosis compared to the low-risk cluster (**Figure 4E**), and the AUC values were greater that 0.7 from the first year to the fifth year (**Figure 4F**). The forest plot of 8 CX3CR1-related IIGs is shown as **Figure 4G**. We also compared the infiltration of activated NK cells and M0 macrophages in two clusters. The bar graph shows that the infiltration of activated NK cells was lower in low-risk group, yet the infiltration of M0 cells was higher in low-risk group (**Figure 5A**).

### High Infiltration of Activated NK Cells in BALF Predicted Poor Diagnosis in IPF Patients

**Figure 6:**
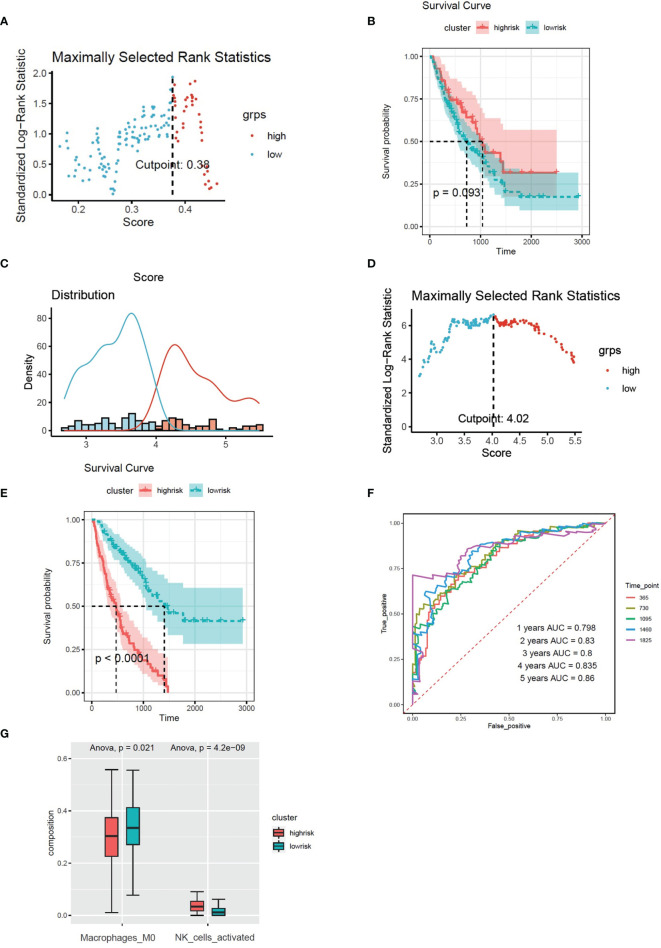
**(A)** The cut-off point with the maximum standard log-rank statistic was marked with a vertical dashed line. **(B)** Kaplan–Meier plot of overall survival in two clusters based on the infiltration of M0 cells. **(C)** Histogram based on maximally selected rank grouping. **(D)** The cut-off point with the maximum standard log-rank statistic was marked with a vertical dashed line. **(E)** Kaplan–Meier plot of overall survival in two clusters based on the combined model (combined risk score). **(F)** The ROC curve in the combined risk models. **(G)** The infiltration of activated NK cells and M0 cells in two clusters based on the combined risk models.

We found that activated NK cells had a positive association with CX3CR1-related genes; however, M0 cells had negative relationship with CX3CR1-related genes (**Figure 3E**). According to the infiltration of activated NK cells, the patients have been divided into high-risk group (40 patients) and low-risk group (136 patients) by the Maximally selected rank method (**Figures 5B, C** **)**. The Kaplan–Meier plot showed that high infiltration of activated NK cells in BALF predicts poor prognosis in IPF patients (**Figure 5D**). The infiltration of activated NK was identified as CX3CR1-related immune cells. We considered the infiltration of activated NK cells as risk score 3. The corresponding ROC plot is shown in **Figure 5E**. Meanwhile, based on the infiltration of M0 cells, the maximally selected rank method has been applied again to divide patients into two clusters (**Figures 5F**, **6A**). There were 56 patients in high-risk group and 120 patients in low-risk group. The Kaplan–Meier analysis demonstrated that high infiltration of M0 cells predicts long survival time than low infiltration group (**Figure 6B**).

### Combined Model Based on Risk Score 1–3 Predicted Poor Diagnosis in IPF Patients

Multivariate Cox regression was applied to build integrated model from risk score 1–3. The combined risk score = risk score1 × (−0.01521) + risk score2 × 1.03705 + risk score3 × (−2.0089). There were 80 patients in the high-risk group and 96 patients in the low-risk group (**Figures 6C, D** **)**. The survival analysis indicated that the high-risk group had poor prognosis than the low-risk group (**Figure 6E**). The AUC values of ROC of the combined model within 1–5 years were greater than 0.7 (**Figure 6F**). We found that the infiltration of activated NK cells was higher in the high-risk group, but the infiltration of M0 cells was lower (**Figure 6G**).

### Preliminary Validation of Clinical Samples

**Figure 7:**
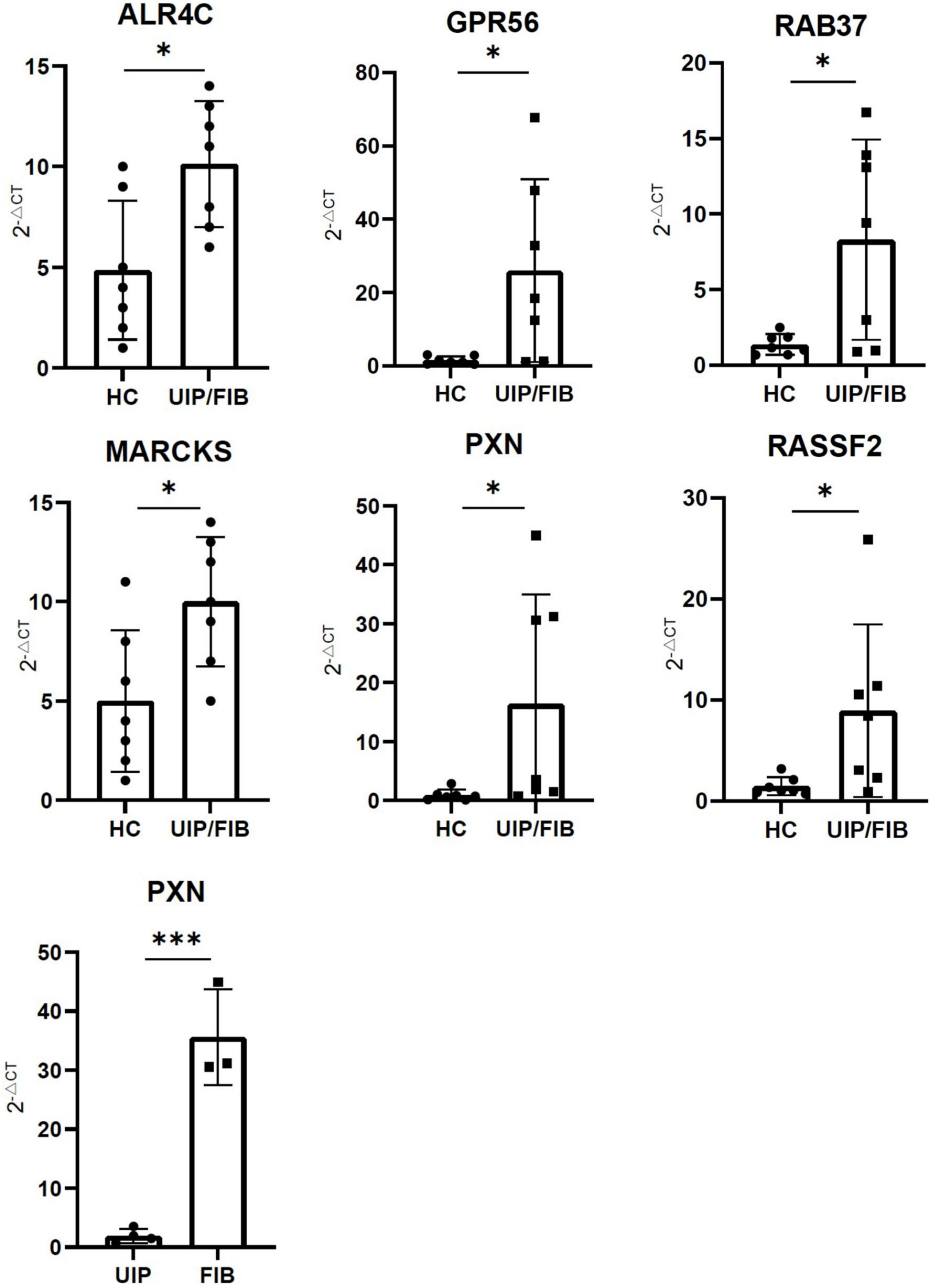
The expression of ALR4C, RAB37, GPR56, MARCKS, PXN, and RASSF2 between healthy control and UIP/FIB patients. The expression of PXN between UIP and FIB patients. *** means P < 0.01, * means P < 0.1.

We found that the expression of ALR4C, RAB37, GPR56, MARCKS, PXN, and RASSF2 were higher in the PBMC of UIP patients and FIB patients compared to healthy controls. The expression of PXN was higher in the PBMC of FIB patients than UIP patients (**Figure 7**).

## Discussion

Due to the lack of effective treatment and poor prognosis of IPF, it is important to establish a personalized treatment plan. Exploring the pathogenesis of IPF is helpful to find better therapeutic targets. To the best of our knowledge, this is the priority study to discuss the effect of CX3CR1 in IPF.

In our study, we found that high expression of CX3CR1 in BALF contributed to the poor prognosis in IPF patients. We identified a group of CX3CR1-related IIGs: ALR4C, RAB37, GPR56, MARCKS, PXN, and RASSF2, which were considered as specific genes in IPF. Through preliminary validation of clinical specimens, we found that the expression of PXN was higher in the PBMC of FIB patients, compared to UIP patients. So, PXN was identified as a disease-specific risk gene in IPF. The infiltration of activated NK cells was influenced by CX3CR1-related IIGs.

Macrophages were considered the major cellular components in BALF (12). A previous study showed that in bleomycin-induced pulmonary fibrosis model, CX3CR1 expression was mainly detected in the majority of macrophages and in a small portion of α-smooth muscle actin (αSMA)-positive cells in the lung (3). This indicated that CX3CR1 was highly expressed in the pulmonary disease. Dvir et al. demonstrated that there was a cluster of CX3CR1^+^ macrophage in fibrotic niche by single-cell analysis, which could promote fibrotic progression in bleomycin-induced murine pulmonary fibrosis model (13). Another study proved that silencing CX3CR1 expression could attenuate pulmonary fibrotic progression in mice (14).

Meanwhile, we found 8 genes related to CX3CR1 and immune cell infiltration. After clinical samples verification, there were 6 genes that have been proven as IPF-specific genes and PXN has been considered as an IPF-specific risk gene. Among the 8 CX3CR1-related IIGs, ALR4C was firstly reported and the coefficient of ALR4C was greater than 0. So, we thought that ALR4C played an anti-fibrotic role in the progression of IPF. RAB37 was also considered as a protective gene in IPF. In the progression of IPF, Wnt signal pathway is activated (15). In addition, many M2 macrophages were activated in the development of IPF. M2 macrophages were significantly associated with fibrotic remodeling of IPF (12). Previous studies showed that when stimulated by RAB37, macrophages went arise to M1 macrophages, while the differentiation to M2 macrophages was inhibited (16). In addition to influence the differentiation of macrophages, RAB37 mediates exocytosis of secreted frizzled-related protein-1 which is an extracellular antagonist of Wnt to inhibit the activation of the Wnt signal pathway (17). Therefore, for our results, we speculated that Rab37 might play a protective role in IPF progression. G protein-coupled receptor 56 (GPR56) mainly functions in cell adhesion and tumor progression. A study elucidated that GPR56 regulated the production of fibronectin and type I collagen and also changed the migratory and invasive capacity of lung fibroblasts (18). In our study, we found that high expression of GPR56 led to poor survival in IPF. Myristoylated alanine-rich C-kinase substrate (MARCKS) is a type of phospholipid binding protein which mainly regulates cell migration, exocytosis, and phagocytosis (19). Previous studies displayed that high expression of MARCKS was detected in IPF patients, and downregulating the expression of MARCKS could decrease the expression of α-SMA and collagens (19). This is consistent with our study and the expression of MARCKS may be impacted by CX3CR1. RASSF2 was considered as a risk gene in our study. However, the role of RASSF2 in fibrotic diseases is rarely reported, and the function of RASSF2 needs to be further studied. In our study, we found that PXN was the disease-specific risk gene in IPF. PXN, also known as paxillin, is a focal adhesion protein, which sends signals from the extracellular matrix, and promotes tumor cell proliferation (20, 21). The activation of the Focal adhesion kinase (FAK)/PXN signaling pathway is important in the development of organ fibrosis. Previous studies demonstrated that the activation of FAK/PXN could drive hepatic stellate cells to differentiate into a fibrogenic phenotype (22). However, suppressing the activation of FAK/PXN could attenuate the development of pulmonary fibrosis in bleomycin-induced pulmonary fibrosis model (23). In our study, we thought that high expression of PXN was one of the risk factors in IPF. This is consistent with previous research that the activation of FAK/PXN leads to the deterioration of organ fibrosis (22). CX3CR1 is closely related to the migration of inflammatory cells. However, PXN is a protein associated with cell surface adhesion. In our study, we found that PXN had a significant association with CX3CR1. Therefore, we speculate whether there is a mutual regulatory relationship between PXN and CX3CR1. Sabine et al. demonstrated that CX3CL1 protected β cells from the adverse effects of TNF-α by restoring the expression and phosphorylation of PXN (24), which was the only study that showed the association between PXN and CX3CR1. The mechanism between FAK/PXN signaling pathway and CX3CL1/CX3CR1 axis needs to be further studied. In our research, we also found that there was a positive correlation between activated NK cells and PXN. A study indicated that PXN was overexpressed in activated NK cells, and inhibition of the expression of PXN decreased secretions of IFN-γ and TNF-α in activated NK cells (25). In summary, PXN was considered as a risk gene in IPF, which might be related to CX3CR1 and activated NK cells.

NK cells mainly function in anti-tumor, antiviral infection, and immune regulation. Previous studies demonstrated that compared to sarcoidosis patients, the infiltration of NK cells in BALF was higher in patients with pulmonary fibrosis (26). Our previous studies showed that high infiltration of NK cells was observed in BALF of IPF patients compared to UIP patients (8). Moreover, we found that high infiltration of activated NK cells contributed to poor prognosis in IPF patients. Meanwhile, we also found that the infiltration of NK cells was correlated to the expression of CX3CR1 and CX3CR1-related IIGs. Additionally, CX3CR1-deficient mice could develop to a very severe experimental autoimmune encephalomyelitis. This was because the deficiency of CX3CR1 could not recruit the migration of NK cells (27). So, we speculated that the infiltration of activated NK cells was regulated by CX3CR1 and CX3CR1-related immune infiltration genes.

There were limitations in our current study. We did not get the desired verification results in BALF, possibly because there were few patients with IPF in our clinical practice, and the clinical samples of BALF from these patients were very difficult to obtain. In our previous study, we found that activated NK cells in the PBMC of the patient migrated to BALF through flow cytometry. So, we thought that PBMC also reflects some disease information of patients. In our verification, we did find that some genes had statistical differences in PBMC. In our next study, we plan to use our collected samples of PBMC for whole genome sequencing. Besides, we did not verify the infiltration of activated NK cells and M0 cells in BALF of IPF patients, and we will do this in the following experiments. Finally, further animal experiments are needed to explore the role of CX3CR1 in IPF in the future.

## Conclusions

We identified a group of genes that were correlated to CX3CR1 and immune cell infiltration in IPF patients. We identified a group of CX3CR1-related IIGs: ALR4C, RAB37, GPR56, MARCKS, PXN, and RASSF2, which were considered as potential genes in for the prognosis of IPF. Through preliminary validation of clinical specimens, we found that the expression of PXN was higher in the PBMC of FIB patients, compared to UIP patients. The combined prognostic model was established to evaluate the survival of IPF patients, which could provide new insights in the therapy and prognosis of IPF patients.

## Data Availability Statement

Publicly available datasets were analyzed in this study. This data can be found here: Gene Expression Omnibus GEO, available at: https://www.ncbi.nlm.nih.gov/geo/query/acc.cgi?acc=GSE70867.

## Ethics Statement

The studies involving human participants were reviewed and approved by The Institutional Review Board of 3rd Xiangya Hospital, Central South University (No. 21028). The patients/participants provided their written informed consent to participate in this study. Written informed consent was obtained from the individual(s) for the publication of any potentially identifiable images or data included in this article.

## Author Contributions

QZ conceived, designed and supervised the study. HC and SC collected and analyzed the data. HC and XL performed the experiments. HC and SC wrote the manuscript. HL and YZ revised the manuscript and edited the language. All authors listed have made a substantial, direct, and intellectual contribution to the work and approved it for publication.

## Funding

This study was supported by grants from the National Natural Science Foundation of China (81700658) and the Hunan Provincial Natural Science Foundation-Outstanding Youth Foundation (2020JJ3058).

## Conflict of Interest

The authors declare that the research was conducted in the absence of any commercial or financial relationships that could be construed as a potential conflict of interest.

## Publisher’s Note

All claims expressed in this article are solely those of the authors and do not necessarily represent those of their affiliated organizations, or those of the publisher, the editors and the reviewers. Any product that may be evaluated in this article, or claim that may be made by its manufacturer, is not guaranteed or endorsed by the publisher.
